# Production study of Fr, Ra and Ac radioactive ion beams at ISOLDE, CERN

**DOI:** 10.1038/s41598-024-60331-z

**Published:** 2024-05-14

**Authors:** E. Jajčišinová, K. Dockx, M. Au, S. Bara, T. E. Cocolios, K. Chrysalidis, G. J. Farooq-Smith, D. V. Fedorov, V. N. Fedosseev, K. T. Flanagan, M. Heines, D. Houngbo, J. D. Johnson, A. Kellerbauer, S. Kraemer, B. A. Marsh, L. Popescu, J. P. Ramos, S. Rothe, M. D. Seliverstov, S. Sels, S. Stegemann, M. Stryjczyk, V. Verelst

**Affiliations:** 1 European Commission, Joint Research Centre (JRC), Karlsruhe, Germany; 2 KU Leuven, Instituut voor Kern- en Stralingsfysica, Leuven, Belgium; 3 SCK CEN, Mol, Belgium; 4 CERN, Geneva 23, Switzerland; 5 Photon Science Institute, Department of Physics and Astronomy, University of Manchester, Manchester, UK; 6 Affiliated with an institute covered by a cooperation agreement with CERN, Geneva, Switzerland; 7 University of Jyvaskyla, Department of Physics, Accelerator laboratory, Jyvaskyla, Finland; 8 Present Address: Department of Oncology Physics, Edinburgh Cancer Centre, Edinburgh, UK; 9 Present Address: SCK CEN, Mol, Belgium

**Keywords:** Experimental nuclear physics, Targeted therapies

## Abstract

The presented paper discusses the production of radioactive ion beams of francium, radium, and actinium from thick uranium carbide (UC_x_) targets at ISOLDE, CERN. This study focuses on the release curves and extractable yields of francium, radium and actinium isotopes. The ion source temperature was varied in order to study the relative contributions of surface and laser ionization to the production of the actinium ion beams. The experimental results are presented in the form of release parameters. Representative extractable yields per $\mu$C are presented for ^222-231^Ac, several Ra and Fr isotopes in the mass ranges 214$\le$*A*$\le$233 and 205$\le$*A*$\le$231 respectively. The release efficiency for several isotopes of each of the studied elements was calculated by comparing their yields to the estimated in-target production rates modeled by CERN-FLUKA. The maximal extraction efficiency of actinium was calculated to be 2.1(6)% for a combination of surface ionization using a Ta ion source and resonant laser ionization using the two-step 438.58 nm, and 424.69 nm scheme.

## Introduction

The field of nuclear medicine is rapidly evolving. Nowadays, the most common radioisotope used for medical diagnostic imaging is ^99m^Tc, representing approximately 80% of all nuclear medicine procedures every year^1^. Radionuclides may also be used for medical treatment. For instance, the $\beta$-particle-emitting ^177^Lu-based drug Lutathera^®^ was approved in 2015 by the Food and Drug Administration and in 2017 by the European Medicines Agency and is today widely used for cancer therapy^2^. Worldwide investigations are ongoing to produce novel radionuclides whose chemical and nuclear properties are optimised for producing radiopharmaceuticals for the treatment of a range of cancer types^3,4^. In the last decade, much interest has been focused on the supply of ^225^Ac for targeted alpha therapy (TAT)^4^. This isotope has been proven to be more efficient than ^177^Lu as it decays by 4 consecutive $\alpha$ particles with energies ranging between 5 - 8.4 MeV. Since they have a higher linear energy transfer (LET) than $\beta$ radiation, more energy is deposited in cancer cells^4^. This is beneficial not only to patients but also to hospital staff due to a shorter radiation range and a smaller dose needed for the same treatment^5^. The demand for this radionuclide is expected to rise every year (unmatched with supply), thus finding a sustainable production way is crucial^6^. Many research groups are trying to find the optimal solution in terms of price, purity, availability and production efficiency^7^. Radionuclide purity is crucial for TAT in order to ensure a high radiolabeling efficiency, to prevent long-lived isotopes from damaging other organs or healthy tissue, and to minimize long-lived waste management in hospitals.

The present work discusses one of the possible production paths for ^225^Ac and other radionuclides by inducing nuclear reactions in thick uranium targets with highly energetic protons in combination with the Isotope Separation On-Line (ISOL) method^8^. Furthermore, this study is very important for other fields that are experimentally studied at ISOLDE. Altogether, Fr, Ra, and Ac isotopes were investigated between *A* = 205-233 in order to characterize their release from the target, extractable yields and their release efficiencies. The release of an isotope from a target can be described as a delay time distribution of the ions after the proton interaction with target. Several different processes influence the isotope release from target and the final extractable yields: in-target production; diffusion to the target surface; desorption from the surface; effusion through the target pores to the target container and transfer line and ionization - surface, electron impact and resonant laser ionization.

Various factors can affect yields extracted at ISOLDE, including laser ionization, target temperature, thermal gradient between ion source, transfer line and target, and ion source temperature itself. Francium (*Z* = 87) belongs to the alkali elements, which ionization potential is typically low (4.07 eV)^9^. Francium is expected to be released rapidly from target. For radium (*Z* = 88), which belongs to the alkali earth elements, the ionization potential is higher (5.28 eV)^10^ but still low enough for surface ionization. However, we expect slower release from target compared to Fr. The surface ionization efficiency for elements with a low ionization potential is high at nominal ion source temperature ($\approx$2000 ^∘^C)^11^. Within the framework developed by Kirchner, a surface ionization efficiency for Fr of 98.1% and for Ra of 31% was calculated (assuming an ion survival of 1 and a number of wall collisions 42)^12^.

The last and most relevant studied element is actinium (*Z* = 89). Actinium has an ionization potential of 5.38 eV^13^, slightly higher compared to Ra, which does not result in efficient ionization on a hot surface, the efficiency being estimated to 3.3% for Ac at 2000 ^∘^C (ion survival of 1, 42 wall collisions)^12^. For elements with a high ionization potential, the use of different ion sources may be required for efficient ionization, such as resonant laser ionization. Furthermore, due to its similar physico-chemical properties to the UC_x_ target material, it is not released from the target easily. Diffusion and effusion from target grains take much longer compared to an alkali element like Fr. Based on these properties, it is reasonable to expect that the release from the target will take longer for Ac than Ra or Fr. Release properties and extractable yields were therefore systematically measured in this experiment to gain a global understanding of the production of those beams, particularly as this was the first laser-ionization of Ac beams at the ISOLDE radioactive ion beam facility.

## Experimental details

The measurements were performed in two campaigns of the IS637 experiment at the CERN-ISOLDE radioactive ion beam facility in Geneva, Switzerland. During both of them, the target was made from depleted uranium (0.29% ^235^U) surrounded by a graphite insert (UC_x_) and placed inside a tantalum container (July 2018 target #658, November 2018 target #638). These targets weigh 100 g with pill diameter of 17 mm. This specific thickness is chosen to achieve an optimal energy loss (about 100 MeV) inside the target instead of stopping the beam completely^14^. Protons accelerated to 1.4 GeV by the Proton Synchrotron Booster (PSB) were delivered to the ISOLDE target with up to 2 $\mu$A intensity. Nuclear reactions induced by these highly energetic protons (mostly fission, spallation and fragmentation) yield numerous radionuclides across the nuclide chart. The nuclear recoils are stopped in the target that is maintained at a temperature of around 2000 ^∘^C through resistive heating. The isotopes undergo diffusion to the target grain boundaries and then effusion through the target container to the ion source. Here, they are ionized and accelerated to an energy between 30–60 keV, separated according to their mass-to-charge ratio through the homogeneous magnetic field of a dipole magnet, and finally delivered to an experimental setup^15^.

The first part used the ISOLDE High Resolution Separator (HRS) made of 2 dipole separator magnets with resolving power of approx. 7000 to separate the beam^16^. Surface ionized beams of Fr and Ra were then delivered to the Alpha Setup (ASET) chamber where alpha spectroscopy of the implanted beam was performed. This setup consists of two silicon detectors: an annular (partially depleted silicon surface barrier - Ortec C Series) detector with active area of 450 mm^2^ and 6 mm diameter hole through which the ion beam passes before implantation onto the foil, and a full (partially depleted Passivated implanted planar silicon - Canberra PD Series) detector with active area of 300 mm^2^ placed behind the foil holder. The geometric efficiency of annular detector is 25(5)% (4 mm from ladder) and that of full detector 36(5)% (3 mm from ladder). Nine 20 $\mu$g/cm^2^-thick carbon foils were placed on a ladder to collect the radioactive ion beam as well as to remove the accumulated radioactivity between measurements. The data were collected by a digital acquisition system based on a CAEN V1724 module and read out by the MIDAS software from Daresbury (UK), enabling event-by-event data collection for offline analysis. This combination of HRS and ASET is suitable for selection of an ion beam at desired mass-to-charge ratio and identification of its isobaric composition by the detection of their decay products. This setup was used to study radioactive isotopes of surface-ionized francium, radium and partially actinium through their $\alpha$ and $\beta$ decays, though actinium was also observed as a decay product.

The second part focused on actinium isotopes. The beam was separated by the ISOLDE General Purpose Separator (GPS) featuring a single dipole magnet with mass resolving power of approx. 2400^16^. The actinium beam was produced with the Resonance Ionization Laser Ion Source (RILIS)^17^. This combination provided element selectivity of resonant laser ionization combined with m/q selection of ions to evaluate production of laser-ionized products vs surface-ionized ones. Resonance laser ionization enhances ionization of the element of interest by resonance excitation of its specific atomic levels, which are unique for each element. The isotopes were then detected with either a Faraday cup for ion beam current measurements, or using the ASET in the same configuration as for the first part. For actinium, only laser ionization resulted in a measurable ion yield. In the two-step process of laser ionization, the valence electron was promoted from the atomic ground state 6d7s^2^ ^2^D_3/2_ to the 6d7s7p ^4^P^∘^_5/2_ level with a laser at 438.58 nm, and then a subsequent excitation to an autoionizing state with a laser at 424.69 nm^18^. By turning on and off the laser beams it was possible to easily switch between different ionization mechanisms and thus to investigate their impact on the radioactive ion beam production. The characteristics of produced ion beams were studied by measuring the ion current using a Faraday cup (FC) in dependence of the mass and ion source temperature.

## Effect of ion source temperature and laser ionization on ^227^Ac production

**Figure 1:**
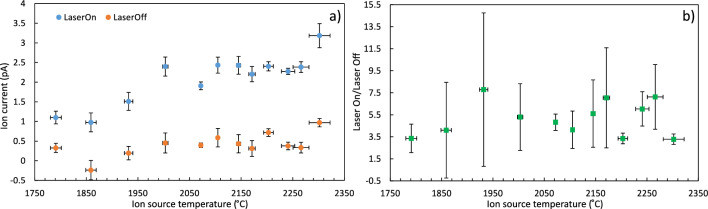
Ion current measurements of the ion beam intensity with mass-to-charge ratio m/q = 227. (**a**) Influence of the ion source temperature on extracted laser- and surface ionized ion beams. The data marked as “Laser On” correspond to a laser enhancement of ^227^Ac. Hence the data marked as “Laser Off” represents surface-ionized species, which are mostly ^227^Fr and ^227^Ra. (**b**) Ratio of total ion beam intensity when “Laser On” relative to the total ion beam intensity when “Laser Off” on mass m/q = 227. Negative currents are caused by the subtraction of the Faraday cup offset. Within the error bar, the ion current nevertheless remains positive.

The radioactive ion beam transmitted through the mass separators contains species with the same mass-to-charge ratio. Complementary use of resonance laser ionization allowed us to study an enhancement of a particular element, in our case Ac. The impact of ion source temperature and laser ionization of actinium was studied on mass *A* = 227 during the second part of the campaign using ASET in collaboration with RILIS. All the measurements were performed with the target held at 2000 ^∘^C. The ion source temperature can be changed indirectly by changing the current applied for the resistive heating, and the temperature is derived from a calibration performed prior to the experiment for each target-ion source unit. In this experiment the ion source temperature was varied from 1790 to 2300 ^∘^C and the effect of blocking and not blocking the first-step laser was investigated by ion current detected in FC. Bunches of $3\times 10^{13}$ protons were delivered in 2.4 $\mu$s duration every 4.8 s^19^. The results are presented in Fig. 1a. Each of these points represent a single measurement on a specific ion source heating temperature. An offset FC reading is subtracted from both data sets, as measured when the ion beam is blocked, which explains why one point scatters in the negative.

One can observe that the enhancement of Ac production by laser ionization is more significant in comparison with the changes in the ion source temperature. The ratio between actinium and other elements remains stable despite the rise in extraction of pure actinium, as shown in Fig. 1b. This means that functioning of both laser and surface ion sources has very similar temperature dependency and lowering the ion source temperature does not provide advantages towards the actinium yield and ion beam purity. From this analysis we see that while long-lived ^227^Ac extraction is enhanced with higher ion source temperatures, this effect is minimal for temperatures beyond 2000 ^∘^C (see Fig. 1). It is critical to find a suitable target and ion source temperature as it affects the production rate and lifespan of a target unit. Furthermore, we can conclude that the fractional production of ^227^Ac increases with temperature although with respect to the isobaric ion beam intensity it remains unchanged.

## Release curves and yields

### Release parameters

The yield and release of many isotopes from ISOLDE targets have been examined in previous years^11,20,21^. It has been reported that for francium with moderate half-lives ($\le$ s), the release from UC_x_ target material is quite similar to ThC_x_ targets^11^. Previous studies of Ra release curves showed a difference in their time structures depending on target material as they were affected by the presence of Fr contamination^20^.

In this experiment, release measurements were performed by studying various isotopes of Fr, Ra and Ac using ASET during both experimental campaigns. This multi-isotope approach enabled us to evaluate the extracted yield of several isotopes of each element as a function of their half-life. The release parameters can be then derived by comparing these yields. The release of different isotopes of the same element should be the same after disentangling the isotope effect in diffusion, differences in effusion speed of atoms in between interactions with container surfaces. This makes some isotopes easier to study than those that are heavily influenced by other factors (e.g. in-target feeding, short half-lives). Since some of the isotopes are long-lived, they may have lower counting statistics as compared to their shorter-lived siblings when monitored via their characteristic decay, but if sufficiently produced, they may be directly measured in a FC as an ion beam current which may still contain isobaric contaminants.

For a given isotope, the release function $P_{i}(t)$ represents the probability density for an atom generated at *t* = 0 to be released at time *t*. The typical release curve shape can be described by four empirical parameters: the sharp rise $\lambda _{r}$, followed by a gradual drop $\lambda _{f}$, ending with a slow tail $\lambda _{s}$ of the function and a parameter $\alpha$ that accounts for the weight between $\lambda _{f}$ and $\lambda _{s}$. These parameters are influenced by sticking time, diffusion, effusion time and other effects^22^. For fitting the release curve data, adding two more parameters, namely a constant $c_{off}$ and time offset $t_{off}$, was necessary for an accurate description. The parameter $t^{*}$ is obtained as $t-t_{off}$. The relation between them and the $P_{i}$ function is as follows^19^:

1 $$\begin{aligned} P_{i}(t^{*},\lambda _{r},\lambda _{f},\lambda _{s},\alpha ) = c_{off}\frac{( 1-e^{-\lambda _{r}t^{*}})}{Norm}\left[ \alpha e^{-\lambda _{f}t^{*}} +(1-\alpha )e^{-\lambda _{s}t^{*}}\right] , \end{aligned}$$

with *Norm* being a normalization factor defined as:

2 $$\begin{aligned} Norm = \alpha \left( \frac{1}{\lambda _{f}}-\frac{1}{\lambda _{f}+\lambda _{r}}\right) +(1-\alpha )\left( \frac{1}{\lambda _{s}}-\frac{1}{\lambda _{s}+\lambda _{r}}\right) . \end{aligned}$$

The parameters can be expressed as times for easier comparison, namely $t = ln(2)/\lambda$. Once the release parameters of all three elements are known for the reference target temperature of 2000 ^∘^C, one can use them to predict the extractable yield of any isotope of that element^19^.

**Figure 2:**
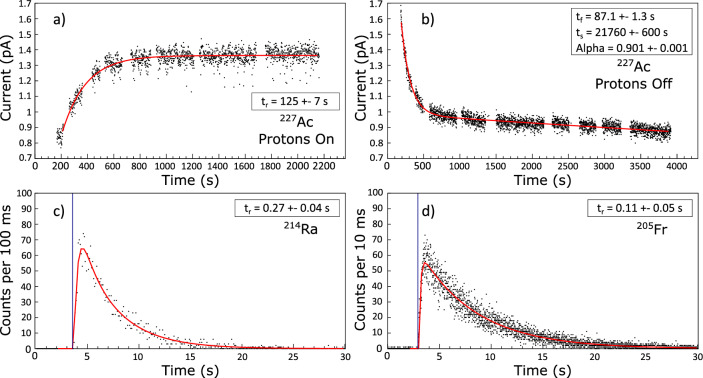
Release curves for Ac, Ra and Fr at 2000 ^∘^C. (**a**) Saturation of ^227^Ac production (proton beam On). Data obtained as ion current on FC. (**b**) Release of ^227^Ac, proton beam Off after saturation. Data obtained as ion current on FC. (**c**) Release curve of ^214^Ra. Data obtained as $\alpha$ particles evolution in time. (**d**) Release curve of ^205^Fr. Data obtained as $\alpha$ particles evolution in time. The blue vertical line represent the proton pulse. The red line represents the fit according to Eq. (1).

The release parameters were obtained for ^205^Fr (T_1/2_ = 3.9 s), ^214^Ra (T_1/2_ = 2.438 s) from the analysis of $\alpha$ particle count rate from the implanted beam reaching the ASET detectors while for ^227^Ac (T_1/2_ = 21.772 y) from the ion beam current measured with the FC following proton impact with the target. Due to the short half-life of the Fr and Ra isotopes, the determination of the $\lambda _{f}$, $\lambda _{s}$ and $\alpha$ parameters was not possible. Additionally, during the data analysis, a strong correlation between the three previously mentioned parameters was observed. To resolve this issue, a series of Monte Carlo simulations were performed to obtain missing parameters. However, due to high interdependence, it was not possible to precisely establish all four parameters for Fr and Ra. Release curves obtained with this analysis are shown in Fig. 2. The respective values are stated in Table 1.

**Table 1:** Release function parameters of Fr, Ra and Ac.

	$t_{r} (s)$	$t_{f} (s)$	$t_{s} (s)$	$\alpha$
Actinium	125(7)	87.1(13)	21760(600)	0.901(1)
Radium	0.27(4)	–	–	–
Francium	0.11(5)	–	–	–

The release parameters of Fr from UC_x_ at 2000 ^∘^C are available in the literature. From A.H.M. Evensen et al.^20^ we can see only qualitatively that the rise parameter of the francium release function from uranium carbide target is in the same order of magnitude as our result. An additional rise time parameter value for Fr is reported by T.E. Cocolios^23^ equal to $t_{r}$ = 0.15 s which is in a good agreement with our obtained value $t_{r}$ = 0.11(5) s.

As expected, the difference in release properties between elements is substantial. While Ac has the longest rise time parameter, Ra and Fr are orders of magnitude shorter, with Fr being the shortest. These parameters indicate that studies of short-lived isotopes from UC_x_ at 2000 ^∘^C with half-lives shorter than their rise time may be challenging. Due to the short half-life of studied francium and radium isotopes, it was not possible to precisely determine the values as the tail was dominated by the decay curve. To extract these values it is necessary to obtain a pure beam of a selected isotope with a half-life in the medium time range.

### Yields

**Figure 3:**
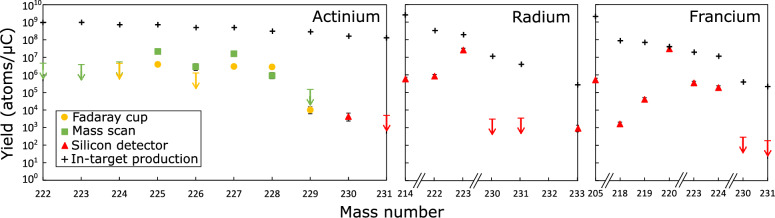
Extracted yields of Ac, Ra and Fr from the UC_x_ target at $\approx$2000 ^∘^C. In-target production yields (+) obtained from the ISOLDE Yield Database^25^ were obtained based on the FLUKA-CERN simulation code^26,27^. Arrows represent upper limits.

Yield describes the number of particles produced in target or the number of particles that can be extracted (extractable yields). The isotope related yields are expressed in units of [1/$\mu$C]. These depend upon the in-target production and on the release properties, as well as the half-life of each isotope:

3 $$\begin{aligned} Y_{i} = \frac{N_{0i}}{N_{p}}\cdot 6.2\times 10^{12}\int _{0}^{\infty }P_{i}(t,\lambda _{r},\lambda _{f},\lambda _{s},\alpha )\cdot e^{-\lambda _{i}t}\,dt, \end{aligned}$$

where $\lambda _{i}$ is the isotope decay constant, $N_{0i}$ is the number of ionized isotopes created by one proton pulse of $N_{p}$ protons^19^. The data available from the present study for the yield analysis originate from the following sources: FC currents (computed average), mass scans and the silicon detectors (release curve integral). Data from FC and mass scans are separated as the mass scan data originates from continuous change of the dipole magnet field other a range corresponding to ion beams at multiple mass-to-charge ratios while FC data are readouts of the average ion beam current over several minutes in a single measurement on the selected mass-to-charge value. The yield values of Ac from FC and mass scans are obtained after offset subtraction and computing the difference between laser on and laser off measurement. This subtraction provides us information about the Ac yields without the isobaric background. Alpha decay offers a characteristic signature that allowed to identify each isotope based on their $\alpha$ energies and on the detected daughters. However, for $\beta$ decay, it was necessary to consider the full combination of implantation and decay and use the Bateman equations to reconstruct the information^24^. This approach was crucial in the *A* = 230,231,233 where Fr, Ra and Ac are in a one chain of $\beta$ decays. Due to selective laser ionization of Ac isotopes, it was possible to distinguish Ac yield from Fr and Ra isobars. The resulting yields were corrected for ion beam transport efficiency (25(5)%) from the focal plane of the mass separator, corrected for the detection efficiency of each technique used, and normalized to obtain the values per $\mu$C. The yields presented in this paper are extractable yields of Fr, Ra and Ac produced at ISOLDE, using UC_x_ target operated at typical temperature conditions 2000 ^∘^C (see Table 2 and Fig. 3).

**Table 2:** Extracted Ac, Ra and Fr yields obtained using FC (marked with $\dagger$), mass scan and silicon detector measurements.

A	T_1/2_	Yield	A	T_1/2_	Yield	A	T_1/2_	Yield
Actinium			Radium			Francium		
222	64 s	$<4.8\times 10^{6}$	214	2.438 s	$6.3(12)\times 10^{5}$	205	3.9 s	$5.5(10)\times 10^{5}$
223	2.1 m	$<3.9\times 10^{6}$	222	38 s	$8.3(15)\times 10^{5}$	218	1.1 ms	$1.9(3)\times 10^{3}$
224	2.78 h	$<4.5\times 10^{6}$ $\dagger$	223	11.43 d	$2.6(6)\times 10^{7}$	219	20 ms	$5.0(9)\times 10^{4}$
224	2.78 h	$<5.5\times 10^{6}$	230	93 m	$<5.9\times 10^{3}$	220	27.4 s	$4.3(8)\times 10^{6}$
225	9.92 d	$4.3(11)\times 10^{6}$ $\dagger$	231	104 s	$<4.5\times 10^{2}$	223	22 m	$4.5(8)\times 10^{5}$
225	9.92 d	$2.3(4)\times 10^{7}$	233	30 s	$9.2(17)\times 10^{2}$	224	3.33 m	$2.4(4)\times 10^{5}$
226	29.37 h	$<1.3\times 10^{6}$ $\dagger$				230	19.1 s	$<2.9\times 10^{2}$
226	29.37 h	$3.1(13)\times 10^{6}$				231	17.6 s	$<1.8\times 10^{2}$
227	21.772 y	$3.3(7)\times 10^{6}$ $\dagger$						
227	21.772 y	$1.7(4)\times 10^{7}$						
228	6.15 h	$3.0(6)\times 10^{6}$ $\dagger$						
228	6.15 h	$9.8(38)\times 10^{5}$						
229	62.7 m	$1.1(5)\times 10^{4}$ $\dagger$						
229	62.7 m	$<1.5\times 10^{5}$						
230	122 s	$4.5(22)\times 10^{3}$						
231	7.5 m	$<5\times 10^{3}$						

The values marked with < represent upper limits.

Multiple approaches could be used to improve the extracted yields from this type of target and this production method. One of them is based on the geometry and temperature profile of the ion source. It is known that the temperature distribution inside the transfer line and ion source features colder spots, which has a strong impact on the ionization or can condensate the transiting atoms. In case of longer irradiation and higher intensity, the ion source faces challenges such as saturation and its inability to keep the temperature constant along the tube^28^. Another way of increasing the extractable yield is by using molecular ion beams. Extraction of radioisotopes from the target in a molecular form can provide a solution to the slow release of actinium but only if effusion time is greater than the diffusion time. Furthermore, the additional mass shift of the molecule helps with the suppression of isobaric contaminants as some elements in a molecule cannot form an ion in certain conditions^29^. An alternative way to increase the extractable production yield of some isotopes is by changing the target material. It was shown in the past that thorium-based targets provide 10-20 times higher cross sections for ^225^Ac and can handle higher primary beam current^7^. Additional option to increase the total production of an isotope is by collecting its precursors. In specific cases isobars may not be considered as contaminants. For instance, at mass *A* = 225, ^225^Fr decays to ^225^Ra, which can contribute to the ^225^Ac production. Thus, it is important to study the yields of all these elements to fully assess the production capability of the isotope of interest, here ^225^Ac.

### Release efficiency

The release efficiency $\epsilon _{tot}$ for each isotope could be determined from the obtained extracted yields. Release efficiency is defined as a ratio between extracted yield to the in-target produced yields. Release efficiencies obtained from this work were calculated using the data of the in-target produced yields from the ISOLDE Yield Database^25^ based on FLUKA-CERN simulations^26,27^. Those simulations were made using $3\times 10^8$ primaries and emulate a steady-state behavior in the target, which enables both direct production and feeding via $\alpha$ or $\beta$ decay. However, it does not consider that some elements, like francium, might be released faster than this steady state might be reached. This may substantially affect the balance of some isotopes, and thus of the trends in release efficiencies, as will be discussed for individual cases.

The efficiencies correspond to the combined efficiency arising from all the processes that the isotope undergoes: diffusion through the target material, desorption from the surface, effusion to the ion source, finally ionization, extraction and others. They depend on the chemistry of each element, on the half-life of each isotope, and are further affected, directly or indirectly, by the temperature of the target and ion source.

**Figure 4:**
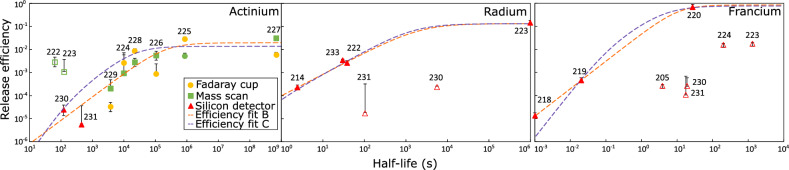
Release efficiency of Ac, Ra and Fr radioisotopes from a UC_x_ target at $\approx$2000 ^∘^C. In-target production obtained from simulation based on the FLUKA-CERN code^25–27^. The empty symbols represent points that are excluded from the fits (more information in text).

The release efficiency is shown as a function of the isotope half-life in Fig. 4. The data were then fitted with two models (more details are given in^21^). First we fitted the actinium release efficiency with the same model as used for release curves (Model C in A. E. Barzakh et al.^21^) with the fixed parameters obtained from our release analysis. This model C is defined as:

4 $$\begin{aligned} \epsilon _{tot}(T_{1/2}) = \epsilon _{extr}\frac{\lambda _{s}(\lambda _{r}+\lambda _{s})}{(\lambda +\lambda _{s})(\lambda +\lambda _{s}+\lambda _{r})}, \end{aligned}$$

where $\lambda = ln(2)/T_{1/2}$. This fit resulted in maximal extraction efficiency $\epsilon _{extr}$ = 1.5(9)%. Better description of our actinium experimental data was achieved with the function defined as model B^21^:

5 $$\begin{aligned} \epsilon _{tot}(T_{1/2}) = \epsilon _{extr}\frac{1}{1+\left( \frac{T_{1/2}}{t_{0}}\right) ^{-\gamma }}, \end{aligned}$$

where $\epsilon _{extr}$ represents maximal extraction efficiency for long-lived isotopes, $t_{0}$ is typical time for diffusion + effusion, and $\gamma$ is the power-function parameter of loss due to release inability for short-lived isotopes^11^. The obtained maximal extraction efficiency and free parameters for Ac are $\epsilon _{extr}$ = 2.1(6)%, $t_{0}$ = $2.0(22)\times 10^{5}$ s, $\gamma$ = 0.9(11). Note that the models 4 and 5 are substantially different however, it can be noted that the time parameter $t_0$ found here and $t_s$ reported in Table 1 are both very long, of the order of $10^4-10^5$ s, which qualitatively highlights the slow release of the actinium from UC_x_. These parameters (see Table 3) may be used to extrapolate the yield of other isotopes of actinium that can be of interest for research.

We can see that the behavior of actinium isotopes can be described by the aforementioned three-parameter model (Eq. 5). The hollow Ac points (^222^Ac, ^223^Ac) were excluded from this fit as their values are influenced by in-target decay of precursors (^226^Pa and ^227^Pa respectively) and the values are quoted as upper limits only. The behavior of radium and francium (middle and right panel) can be examined with both models as well if we exclude the hollow points from the fit and fix the rise parameter $\lambda _{r}$ in Eq. (4) using the values reported in Table 1. An additional characteristic appears in the francium isotopic chain. From the right panel of Fig. 4, the full points seem to follow their own release trend. These values of francium ^218^Fr, ^219^Fr, ^220^Fr originate from the same conditions (same campaign, target material, analysis technique) and all of them are constantly fed by in-target decay of their Ac precursors and therefore we used them as reference for the fit while keeping the rise parameter from Eq. (4) fixed. Hollow points ^230^Fr, ^231^Fr and ^230^Ra, ^231^Ra deviate from the trend as their production yield is highly influenced by their chain of $\beta$ decays starting from Fr to Ra and then Ac isobars. The yields obtained for these masses are limited by the analysis technique used, more precisely by deconvolution of $\beta$ particles time distribution. Lower efficiency of ^223^Fr, ^224^Fr can be explained by the origin of the data as the detection threshold was set too high and some of the events were not recorded. A very low release efficiency of ^205^Fr could be explained from the FLUKA simulated production yield used as a reference. This model predicts a production yield that is a factor of 42 times higher than predicted by ABRABLA, highlighting the challenge of accurate predictions of an isotope originating from spallation reactions so far from the target material.

**Table 3:** Results of Ac, Ra and Fr release efficiencies fits presented in Fig. 4.

	Eq. (4)			Eq. (5)		
	$t_{r} (s)$	$t_{s} (s)$	$\epsilon _{extr} \%$	$\gamma$	$t_{0} (s)$	$\epsilon _{extr} \%$
Actinium	125(7)	21760(600)	1.5(9)	0.97(103)	2.0(22)$\times 10^{5}$	2.1(6)
Radium	0.27(4)	1550(335)	13.7(25)	0.94(9)	2230(1450)	13.8(25)
Francium	0.11(5)	5.2(15)	91(18)	1.21(9)	10.8(87)	101(35)

From this analysis we obtained maximal extraction efficiency of Ac equal to 2.1(6)%. At the offline CERN MEDICIS facility, laser ionization of actinium originating from an actinium nitrate mass marker deposited on a rhenium surface and on a ThO_2_ matrix reached 15.1(6)%^30^. The conditions were substantially different between those offline measurements and the experiment reported here, in particular the absence of diffusion through the target material. The chemistry of the target is definitely a crucial aspect, as the present work used a carbide target, compared to the actinium nitrate mass marker or the use of an oxide target. Moreover, the ThO_2_ study at MEDICIS required very high operational temperatures in excess of 2200 ^∘^ C to release the Ac. Another difference is in the use of rhenium ion source instead of tantalum and in laser ionization scheme. The first step laser was the same but two second transitions were used with laser set to 424.70 nm and 456.15 nm, the latter was shown to be more efficient by $\approx$50%. Furthermore we showed a good agreement in release efficiency parameters of Ra and Fr to the theory in^11^. We can see that $t_{0}$ parameter for Ra was 1.4(53)$\times 10^3$ s in comparison to our value 2.2(15)$\times 10^3$ s. For Fr they obtained $t_{0}$ = 17(8) s in comparison to our value 10.8(87) s.

## Conclusions

To summarize the presented work, the relative population of elements in the ion beam cannot be changed only by tuning the ion source temperature. The impact of laser ionization is relevant as it results in an increase in the production rate of the element of interest, namely Ac. Furthermore, maintaining the ion source temperature at 2000 ^∘^C or above is essential for production of Ac using RILIS.

From the study of element release from the UC_x_ target at 2000 ^∘^C, we confirmed that the release time of francium and radium isotopes is orders of magnitude faster compared to actinium. The analysis of extractable yields brought an insight into Fr, Ra and Ac production. Furthermore, in this work the production yield of ^225^Ac from UC_x_ reaches approximately $2.3\times 10^7$ particles (19 Bq) of ^225^Ac per $\mu$C. This means that ^225^Ac can be produced at $\approx$103 MBq in target during a 1-day irradiation while $\approx$3.1 MBq can be extracted (assuming 2 $\mu$A proton). It is important to realize, that production is, after mass separation and collection of the sample, supplemented by the decay of produced ^225^Fr (T_1/2_ = 4.0 m) and ^225^Ra (T_1/2_=14.9 days) which act as ^225^Ac generators after purification^31^. The release efficiency for Ac isotopes is relatively low due to the nonvolatile behavior of Ac. Furthermore, the maximal extraction efficiency of actinium from this work was established as 2.1(6)%. This efficiency can be improved by changing the laser ionization scheme^30^. The maximal extraction efficiency of Ra and Fr was established as 13.8(25)% and 101(35)% respectively, in line with expectations based on their chemical nature.

The present study provides a better insight into the production of radionuclides relevant not only for nuclear medicine but also for other ISOLDE experiments^32^. It highlights in particular where improvements on the radioactive ion beam production can be made.

## Data Availability

The datasets generated during and/or analysed during the current study are available from the corresponding author on reasonable request.
